# Effects of Transcutaneous Electrical Acustimulation on Refractory Gastroesophageal Reflux Disease

**DOI:** 10.1155/2016/8246171

**Published:** 2016-08-28

**Authors:** Li-na Meng, Shanshan Chen, Jiande D. Z. Chen, Hai-feng Jin, Bin Lu

**Affiliations:** ^1^Key Laboratory of Digestive Pathophysiology of Zhejiang Province, Division of Gastroenterology, The First Affiliated Hospital of Zhejiang Chinese Medical University, Hangzhou 310006, China; ^2^Ningbo Pace Translational Medical Research Center, Beilun, Ningbo 315043, China; ^3^Division of Gastroenterology and Hepatology, Johns Hopkins University, Baltimore, MD 21224, USA

## Abstract

*Objective*. To investigate effects and possible mechanisms of transcutaneous electrical acustimulation (TEA) performed by a wearable watch-size stimulator for refractory gastroesophageal reflux disease (RGERD).* Methods*. Twenty patients diagnosed as RGERD were enrolled in the study and randomly divided into four groups: esomeprazole group (Group A), esomeprazole combined with TEA group (Group B), esomeprazole combined with sham-TEA group (Group C), and esomeprazole combined with domperidone group (Group D). HRM and 24 h pH-impedance monitoring and GerdQ score were used to measure related indexes before and after treatment.* Results*. (1) TEA significantly increased LESP, compared with PPI treatment only or PPI plus sham-TEA. After pairwise comparison, LESP of Group B was increased more than Group A (*P* = 0.008) or Group C (*P* = 0.021). (2) PPI plus TEA decreased not only the number of acid reflux episodes but also the number of weak acid reflux episodes (*P* = 0.005). (3) Heartburn and reflux symptoms were improved more with PPI + TEA than with PPI treatment only or PPI plus sham-TEA (GerdQ scores, *P* = 0.001).* Conclusion*. TEA can improve symptoms in RGERD patients by increasing LESP and decreasing events of weak acid reflux and acid reflux; addition of TEA to esomeprazole significantly enhances the effect of TEA.

## 1. Introduction

Gastroesophageal reflux disease (GERD) is defined as a condition that appears when the reflux of stomach contents causes troublesome symptoms and/or complications. The incidence of the disease is gradually increased. Although the emergence of PPI therapy for GERD has greatly helped, there are still some patients whose symptoms cannot be alleviated after PPI therapy. A concept “refractory gastroesophageal reflux disease” (RGERD) has been proposed. According to 2013 US GERD guidelines, patients who do not respond to a 8–12 weeks' course of acid-suppressive therapy with PPI twice daily and the fact that the GERD associated symptoms still exist and affect the quality of life or GERD associated symptoms' improvements are less than 50% can be diagnosed as RGERD [1].

Doubling proton pump inhibitors (PPIs) dose represents the mainstay of treatment for RGERD [2]. Other nonmedical treatment options including endoscopic therapy and surgical approaches could be considered, according to individual patient characteristics. Transoral incisionless fundoplication (TIF) and Stretta are emerging endoscopic therapies with minimal side-effects [3–6]. Another recent method, LES electrical stimulation therapy (LES-EST), which uses an implanted stimulation system is showing some unique advantages, especially for those with ineffective esophageal motility or aperistalsis or those who are unable to undergo traditional antireflux surgery. However, these promising therapies are all invasive and their long-term efficacy and safety need further evaluation.

Acupuncture has been proved to have a good effect for GERD. Adding acupuncture was more effective when compared with doubling the PPI dose in controlling symptoms in GERD patients who were unresponsive to standard-dose PPI [7]. Electroacupuncture (EA) was found to enhance the gastric peristalsis and accelerate gastric emptying and improve esophageal peristalsis, as delayed gastric emptying has also been shown to contribute to failure of PPI once daily [8, 9].

Currently, transcutaneous electrical acustimulation (TEA), a noninvasive and needleless method that is different from traditional electroacupuncture, has been proposed [10–13]. It can be used at home and administrated daily and even more than one time daily which could make the therapy more effective. TEA was reported to markedly improve dyspepsia symptoms [12]. However, it is unknown whether TEA is effective for RGERD. This present study was designed to investigate effects and possible mechanisms of TEA for RGERD.

## 2. Materials and Methods

### 2.1. Patients

Twenty patients with RGERD seen at the Gastroenterology clinic of The First Affiliated Hospital of Zhejiang Chinese Medical University from February 2013 to June 2014 participated in the study. The protocol was approved by Ethics Committee of The First Affiliated Hospital of Zhejiang Chinese Medical University. All the patients had reflux-related symptoms for at least one year and failed to respond to a 8–12 weeks' course of acid suppression with PPI twice daily.

Inclusion criteria were outpatients at the age of 16–70 years. Exclusion criteria included severe cerebrovascular disease, hepatic or renal failure, and hematopoietic system disease; women during pregnancy and lactation; and peptic ulcers, esophageal or gastric polyps, esophageal cancer, gastric dysplasia, gastric cancer, or previous upper gastrointestinal surgery such as subtotal gastrectomy.

### 2.2. Measurements

*(1) Esophageal High-Resolution Manometry (HRM).* All participants underwent electronic gastroscopy before HRM test (ManoScan 360, Given Imaging/Sierra Scientific Instruments, USA). Manometric data were analyzed by ManoView Analysis (Sierra Scientific Instruments). After fasting for 8 h, esophageal catheter was inserted transnasally after pressure calibration. When the upper esophageal sphincter (UES), esophageal body, esophagogastric junction (EGJ), and gastric pressure were simultaneously displayed on the screen, the catheter was fixed. After being placed in supine position, participants were asked to stop swallowing within 30-s and then swallow boluses of 5 mL of water, which were injected into their mouth by using a syringe [14].

High-resolution manometry (HRM) was used to detect LES resting pressure (<13 mmHg was abnormal) and esophageal motility. According to the Chicago Classification V3.0, esophageal motility disorders include ineffective esophageal motility (IEM) and fragmented peristalsis. IEM is ≥50% ineffective swallows. Ineffective swallows can be failed or weak (distal contractile integral (DCI) < 450 Hg·cm·s). Fragmented peristalsis is large break (>5 cm length) in the 20-mmHg isobaric contour with DCI > 450 Hg·cm·s [14].

*(2) 24 h pH-Impedance Monitoring*. Before treatment, participants stopped using PPI and prokinetics more than one week before 24 h pH-impedance monitoring; after treatment, the test was performed under medication in over 8 h fasting status. Esophageal manometry was performed first to determine the distance from nostrils to lower esophageal sphincter (LES). Then, the pH electrode was placed at 5 cm above LES. pH and impedance data were recorded by a digital data logger (Given Imaging/Sierra Scientific Instruments, USA). Once the pH recording was initiated, participants were instructed to assume normal daily activities and dietary practices. Patients documented in a diary their ingestion, sleep periods, and the occurrence of GERD related symptoms [15].

The analysis was performed for DeMeester score, total reflux episodes, acid reflux episodes (pH ≤ 4), weak acid reflux episodes (4 < pH < 7), and nonacid reflux episodes (pH ≥ 7). The normal range is as follows: DeMeester score ≤ 14.72, acid reflux ≤ 55 times, weak acid reflux ≤ 26 times, nonacid reflux ≤ 1 time, and total reflux episodes ≤ 73 times.

*(3) GERDQ Questionnaire [16]*. Patients were asked to recall symptoms present during the last 7 days and complete the questionnaire which included A: heartburn and regurgitation, B: a pain in the center of the upper stomach and nausea, and C: sleep disturbances and taking additional medication. A + B + C ≥ 8 indicated a positive test.

### 2.3. Study Design

The study was designed as a randomized controlled, single blind, prospective trial. Enrolled patients were randomly allocated into 4 groups and received 4-week treatment.

Group A was as follows: esomeprazole 20 mg, twice daily.

**Figure 1:**
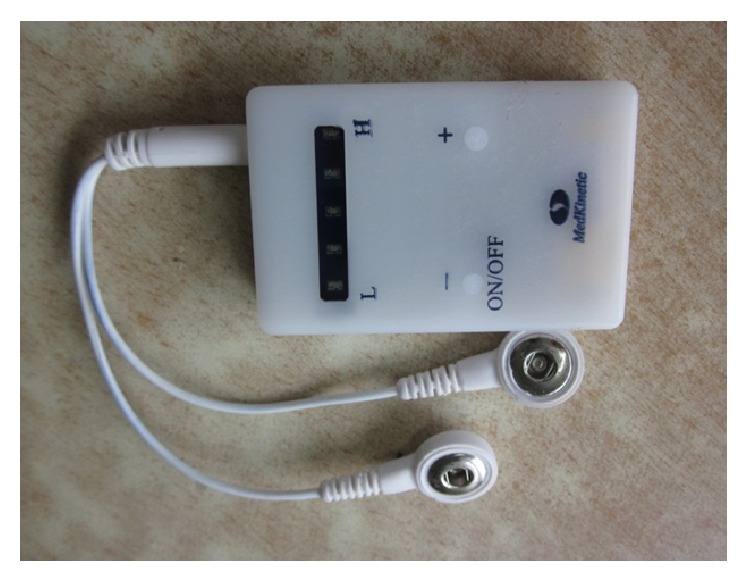
The wearable watch-size stimulator.

Group B was as follows: esomeprazole 20 mg twice daily + TEA (Zusanli, Neiguan) 25 Hz 5 mA 30 min twice daily using a watch-size stimulator (MedKinetic, Ningbo, China). The device is shown in Figure 1.

It can be attached to the arm and leg. The stimulation parameters were programmed via a computer by the investigator. However, the patient was able to adjust the stimulation output current to a level that is sensible but tolerable.

Group C was as follows: esomeprazole 20 mg twice daily + Sham-TEA, 25 Hz 5 Am 30 min, twice daily.

Group D was as follows: esomeprazole 20 mg twice daily + domperidone 10 mg 3 times/day.

 Acupoints are as follows:

1. Zusanli: It is located 5 mm below and lateral to the anterior tubercle of the tibia.
2. Neiguan: It is located 1.5–2.0 cm above the wrist crease between the tendons of the flexor carpi radialis and the palmaris longus.
3. Sham-TEA: It is virtual acupoint, nonchannel noncollateral acupoint 2 cm near Zusanli or Neiguan.

High-resolution manometry (HRM) and 24 h pH-impedance monitoring were used to observe LES relaxing pressure and esophageal clearance capacity and analyze reflux of RGERD patients in each group before and after treatment. GERDQ score was recorded to evaluate the improvement of RGERD symptoms.

### 2.4. Statistical Analysis

SPSS17.0 statistical software (SPSS Inc., Chicago, IL, USA) was used for data analysis. Measurement data are expressed as mean ± standard deviation or median (25th percentile, 75th percentile). Multiple parametric groups were compared using the one-way ANOVA test (normal distribution) or nonparametric test (not normal), pre- and posttreatment were evaluated using paired *t*-test; count data were compared with rate or percentage (%) and *X* ^2^ test was used for comparison between groups. *P* values of 0.05 or less were considered to be statistically significant.

## 3. Results

Twenty patients (11 M, 9 F; mean age: 51.5 ± 13.8 versus 46.7 ± 12.4) were recruited into the study. Among the 20 patients, 7 patients (35%) had hypotensive LESP and 13 patients (65%) had esophageal peristalsis disorders. The median of DeMeester score is 4 (1.1–16.8), while the median of acid reflux, weak acid reflux, nonacid reflux was 11 (4–17), 27 (7–42), and 8 (2–16), respectively.

### 3.1. Effects of TEA on Esophageal Motility

**Figure 2:**
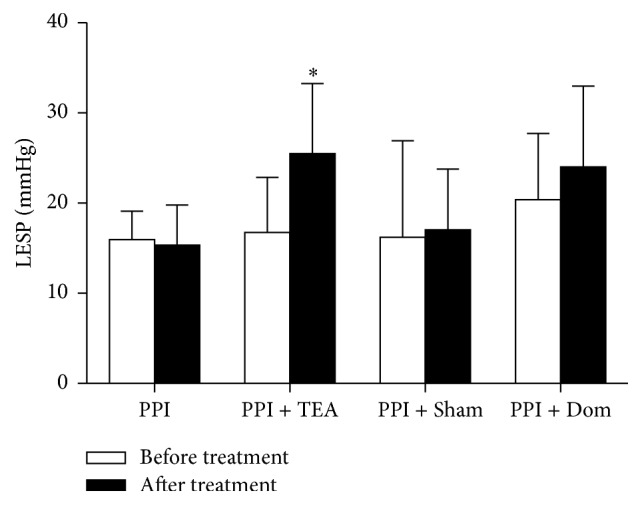
Low esophageal pressure before and after the treatment in different groups. A significant increase was noted only in patients treated with TEA (^*∗*^ *P* = 0.004).

TEA significantly increased LESP, and this increase was not noted in any other group. As shown in Figure 2, a 52.2% increase in LESP can be appreciated in patients treated with PPI + TEA (*t* = 5.91, *P* = 0.004).

The posttreatment changes in LESP among groups were statistically significant (*F* = 3.57, *P* = 0.038); the change in the PPI + TEA group was significantly higher compared to the PPI group (*P* = 0.008) or the PPI + TEA group (*P* = 0.021) but not the PPI + Dom group (*P* > 0.05).

None of the treatments resulted in any significant changes in esophageal contractile activities, such as esophageal motility disorders (IEM) and fragmented peristalsis.

### 3.2. Effects of TEA on Acid and Weak Acid Reflux

**Figure 3:**
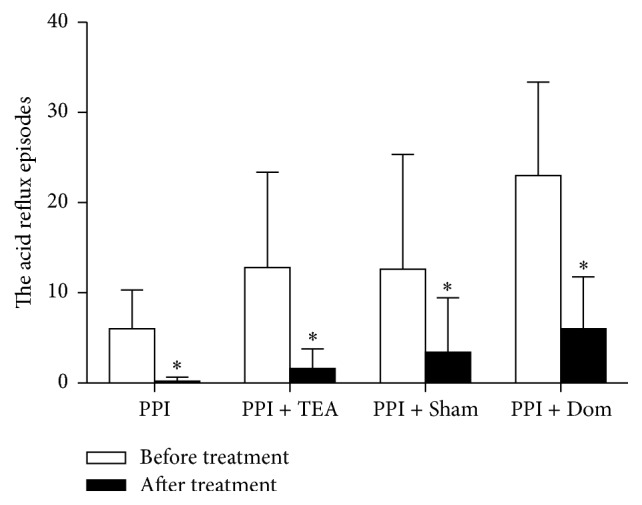
The acid reflux episodes of all the groups decreased after treatment (^*∗*^ *P* < 0.05). The change of the acid reflux episodes was not significantly different among groups (*P* > 0.05).

**Figure 4:**
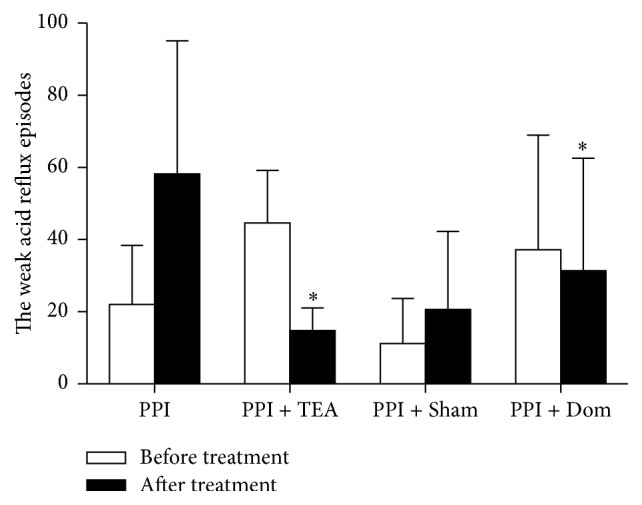
The weak acid reflux episodes of Group B and Group D decreased after treatment (^*∗*^ *P* < 0.05).

While acid reflux was reduced in all groups, weak acid reflux was suppressed only in the group treated with TEA. As shown in Figure 3, the number of acid reflux episodes was significantly reduced in all 4 groups of patients, as PPI was given in all groups. No difference was noted in the potency of acid suppression among the 4 groups. However, a change in weak acid reflux was observed only in the patients treated with TEA (*P* = 0.011 before versus after treatment in Group PPI + TEA, Figure 4). The treatment-induced changes in weak acid reflux were significantly different among the 4 groups (*F* = 6.34, *P* = 0.005). Pairwise comparison revealed that the decrease in weak acid reflux in Group PPI + TEA was significantly higher compared to Group PPI (*P* = 0.001) or Group PPI + Sham (*P* = 0.023) but not Group PPI + Dom (*P* > 0.05).

However, a number of other parameters, including DeMeester score, total reflux events, and nonacid reflux events, were not significantly different before and after any treatment (*P* > 0.05).

### 3.3. Effects of TEA on Symptoms

**Figure 5:**
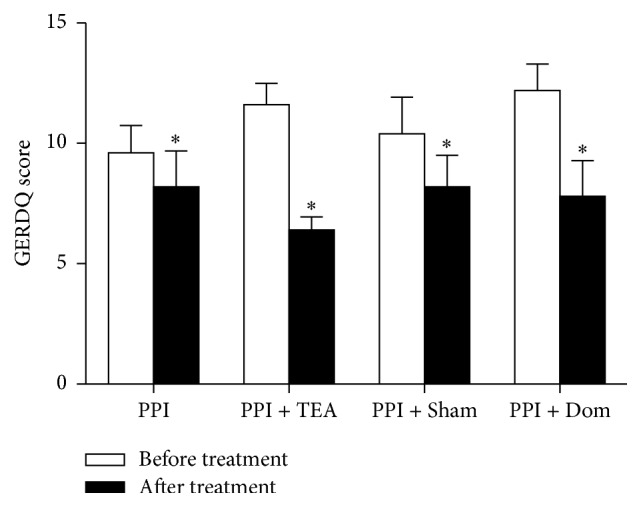
The GERDQ scores of all the groups decreased after treatment (^*∗*^ *P* < 0.05).

All treatments resulted in significant improvement in reflux-related symptoms, reflected as a decrease in the GERDQ score (Figure 5, *P* < 0.05 before versus after treatment for all groups). However, a significant difference was noted in the treatment-induced reduction in the GERDQ scores among the 4 groups (*F* = 7.59, *P* = 0.001); pairwise comparison showed that this decrease in Group PPT + TEA was significantly higher compared to Group PPI (*P* = 0.001) or Group PPI + Sham (*P* = 0.001) but not Group PPI + Dom (*P* > 0.05).

## 4. Discussion

In this study, we found that TEA significantly increased LESP and reduced weak acid reflux. All treatments, especially PPI + TEA, resulted in significant improvement in reflux-related symptoms.

Previous studies have demonstrated that up to 40% of GERD patients had a lack of response to a standard PPI dose once daily [1]. Doubling PPI dose represents the mainstay of treatment for RGERD. Switching to another PPI is another common and cost-effective approach. But it has certain limitations because symptoms may be related to weak acid reflux or nonacid reflux. The decline in antireflux defense mechanisms plays a major role in RGERD patients. Medical treatment has been primarily focused on reducing transient lower esophageal sphincter relaxation rate or attenuating esophageal pain perception using visceral analgesics [6]. Improving LES function and esophageal peristalsis is considered as a potential new therapeutic method.

Acupuncture has been applied for the treatment of various digestive diseases. It was found to enhance gastric peristalsis and accelerate gastric emptying as delayed gastric emptying has also been shown to contribute to failure of PPI once daily [8, 9]. Adding acupuncture to the standard PPT therapy was found to be more effective when compared with doubling the PPI dose in controlling symptoms in GERD patients who were unresponsive to standard-dose PPI [7]. Needleless TEA does not damage the skin and prevent the spread of infection or disease so that is more easily accepted in clinic. Patients can carry instruments with them that make it convenient for their self-management.

In our study, high-resolution manometry (HRM) was used to observe the changes of esophageal motility in static and dynamic way. As the esophagogastric junction (EGJ) is an important barrier to defense against gastroesophageal reflux occurrence, including the LES and crura of diaphragm, the decline of antireflux defense mechanisms especially hypotensive lower esophageal sphincter plays a major role in RGERD patients. A latest research shows that a short-term electrical stimulation of the lower esophageal sphincter increases sphincter pressure in patients with GERD [17]. After TEA, LESP was increased significantly.

In addition, 24-hour pH-impedance monitoring was performed for acid reflux episodes, weak acid reflux episodes, and nonacid reflux episodes. Weak acidic reflux, in large multicenter studies, has been recognized as one of the main mechanisms underlying PPI failure in patients with regurgitation and atypical GERD symptoms [18–20]. Antacids can reduce the number of acid reflux episodes, but the weak acid reflux and nonacid reflux episodes are correspondingly increased. Our results showed that weak acid reflux was suppressed only in the group treated with TEA while acid reflux was reduced in all groups.

The possible mechanism involved in the improvement of reflux with the addition of TEA is believed to be attributed to the increase in the LESP. Although not investigated in the present study, previous findings using similar method of TEA or electroacupuncture at ST36 and/or PC6 led us to speculate the following mechanisms and pathways [10–12]. TEA at ST36/PC6 activates peroneal and median nerves, which leads to the activation of spinal afferent neurons [21]. The spinal neural signal activates neurons in the nuclear tractus solitarius, resulting in enhanced vagal efferent outflow at the dorsal motor nucleus of the vagus [22, 23]. The increased vagal efferent activity stimulates release of acetylcholine at the lower esophageal sphincter, resulting in an increased LESP. In addition to the increase in LESP, other possible mechanisms may involve gastric emptying and gastric accommodation. In patients with functional dyspepsia, TEA using the same method as in this study was shown to accelerate gastric emptying [12] and improve gastric accommodation [10] also mediated via the vagal pathway. Since delayed gastric emptying and impaired gastric accommodation are known to induce gastroesophageal reflux, the improvement in these abnormalities might lead to the improvement of the reflux.

In summary, needleless TEA using a watch-size wearable device may improve reflux-related symptoms in patients with refractory GERD by increasing LESP and reducing weak acid and acid reflux. Large-scale clinical studies are warranted to explore therapeutic role of TEA for refractory GERD.
